# Machine Learning for Authentication and Authorization in IoT: Taxonomy, Challenges and Future Research Direction

**DOI:** 10.3390/s21155122

**Published:** 2021-07-28

**Authors:** Kazi Istiaque Ahmed, Mohammad Tahir, Mohamed Hadi Habaebi, Sian Lun Lau, Abdul Ahad

**Affiliations:** 1 Department of Computing and Information Systems, Sunway University, Petaling Jaya 47500, Selangor, Malaysia; kazi.i@imail.sunway.edu.my (K.I.A.); sianlunl@sunway.edu.my (S.L.L.); abdul.a22@imail.sunway.edu.my (A.A.); 2 IoT & Wireless Communication Protocols Lab, Department of Electrical and Computer Engineering, International Islamic University Malaysia, Jalan Gombak 53100, Selangor, Malaysia; habaebi@iium.edu.my

**Keywords:** Internet of Things, IoT, security, authentication, authorization, machine learning

## Abstract

With the ongoing efforts for widespread Internet of Things (IoT) adoption, one of the key factors hindering the wide acceptance of IoT is security. Securing IoT networks such as the electric power grid or water supply systems has emerged as a major national and global priority. To address the security issue of IoT, several studies are being carried out that involve the use of, but are not limited to, blockchain, artificial intelligence, and edge/fog computing. Authentication and authorization are crucial aspects of the CIA triad to protect the network from malicious parties. However, existing authorization and authentication schemes are not sufficient for handling security, due to the scale of the IoT networks and the resource-constrained nature of devices. In order to overcome challenges due to various constraints of IoT networks, there is a significant interest in using machine learning techniques to assist in the authentication and authorization process for IoT. In this paper, recent advances in authentication and authorization techniques for IoT networks are reviewed. Based on the review, we present a taxonomy of authentication and authorization schemes in IoT focusing on machine learning-based schemes. Using the presented taxonomy, a thorough analysis is provided of the authentication and authorization (AA) security threats and challenges for IoT. Furthermore, various criteria to achieve a high degree of AA resiliency in IoT implementations to enhance IoT security are evaluated. Lastly, a detailed discussion on open issues, challenges, and future research directions is presented for enabling secure communication among IoT nodes.

## 1. Introduction

It is estimated that there will be over 30 billion devices connected to the Internet by the end of 2025 [1]. The reason for such growth is the promise that IoT holds to improve business processes and accelerate growth. IoT represents interconnected physical things that are uniquely identified with any intelligent devices/objects (e.g., sensor) that are, in turn, connected to the Internet in a heterogeneous environment and can generate events [2,3]. Therefore, providing security on IoT nodes to protect boot sequence, management of keys, data protection, secure sessions on communication establishments, reliable hardware/software patches, and monitoring and auditing are crucial for exchanging data among nodes. The sharing of data among IoT nodes directly affects IoT security and is largely based on the trustworthiness of the data and the standard of service rendered. For example, a distributed denial of service (DDoS) attack was originated from millions of IoT devices infected by the Mirai malware. It is a simple program available online, where many IoT devices like closed-circuit cameras and smart-home devices were hacked by malware and used against the servers [4]. This attack highlights the need to design a proper security mechanism for the widespread adoption of IoT and avoid future security breaches resulting in personal data leaks and monetary loss. Authentication and authorization (AA) are the first lines of defence and are intended to restrict actions and operations in IoT environments [5]. AA aims to prevent breaches by enforcing user restrictions, resulting in the exposure of essential resources to unwanted opponents. Authentication, including protection tools to prevent external attacks, such as man in the middle attack [6] and eavesdropping attack [7,8] for the application, are generally customized to tackle different threats at certain network circumstances. However, malicious behaviour is unpredictable and cannot be dealt with before each attack. AA offers security services to counter internal and external threats. To counter these threats, machine learning (ML)-based AA is a promising tool when it comes to building security schemes that learn how to identify and counteract new and existing complex attacks efficiently by repeatedly analyzing the connectivity patterns of nodes following efficient initial authentication, such that any behavioural shifts from valid to suspicious can be observed. Due to resource limitations, it is not feasible to explicitly implement ML techniques on IoT devices. Therefore, robust ML-based AA with feature reduction techniques are expected to minimize resource usage with optimizing accuracy, efficiency, and data availability [9].

### 1.1. Current Issues and Motivation

To enable the widespread adoption of IoT, various security requirements must be addressed [10,11]. Authentication and authorization are crucial security provisions that require an extensive focus from IoT security researchers. At present, most IoT implementation relies on centralized client-server architecture, connecting to the cloud via the Internet. All devices are identified, authenticated, and connected by cloud servers that support vast processing and storage capacities. The communication between IoT devices will have to go through the cloud, even when they are in the vicinity. Such a model is prone to bottlenecks, downtime, and coordinated attacks that might affect the operation of the entire network. The problem is further aggravated due to the resource-constrained nature of IoT devices. The current best security practices cannot be implemented to secure the IoT ecosystem, as they were not designed for resource-constrained devices, leaving billions of devices prone to attack. Traditional approaches work well for different aspects of IoT (e.g., clustering, protocols, applications, data aggregation, services, architectures, resource allocation), including security for now. In the near future, there will be a need for AA schemes that handle access in a distributed manner in a secure and efficient from a large number of IoT nodes in the future. In various implementations, such as remote health care services, energy utilization management, traffic control management, and smart system for parking, home and industry, IoT has become a significant component. In all such implementations, consumers and service providers require the confidentiality of their activity, behaviours, and personal details. IoT must guarantee the end-to-end security and privacy of its users to achieve the confidence of users.

While ML and IoT access management systems are currently significant areas of study, limited literature exists on IoT security based on ML that focuses on authentication and authorization [12]. Nevertheless, owing to the characteristics of IoT nodes, the study also has several limitations in the AA aspect. As we find new IoT threats very frequently, it is important to explore and address various security issues via the most up-to-date and thorough analysis of the ML-based security solution.

Authentication and authorization are significant security issues in IoT, and several research studies have taken place in the domain. In this paper, we focus on general and ML-based authentication schemes for IoT. A summary of the state-of-the-art research leveraging ML algorithms for AA of IoT is presented. Furthermore, we provide a discussion of security concerns related to AA on the supporting layer of IoT.

### 1.2. Authentication and Authorization in IoT

Authentication and authorization are two key components to protect customers and devices on the Internet. It makes these components essential to IoT implementation, because the IoT is simply a device at its most fundamental level, from simple sensors to cars and complicated mobile devices, which connect to share data. Authentication is the device identification process which confirms the validity of the device client ID and makes sure that the ID belongs to that specific device only. Authorization is a way of assessing a node (sensor node or user) that has the permission to access resources, such as reading or writing data, running programs, and controlling actuators. Authorization also covers access denial or revocation, especially for somebody or something malicious. Furthermore, authorization provides a mechanism for linking a specific device (or subject membership group of devices) to individual services.

There are two types of authentication and authorization processes: one for the devices and another for the users. This review study focuses on device authentication and authorization. An excellent example of this is a sensor. Before the communication session starts and the sensory data exchange takes place, device identity and authorization level is set through the AA processes.

### 1.3. Existing Reviews

Several research papers have covered IoT security-related issues. For example, authors in [13] presented an overview of “autonomic security” for IoT threat mitigation strategies. The authors classified these strategies into self-protecting, self-healing, and a hybrid of self-protecting and self-healing. They described the usage against different threats in three layers: perception, communication, processing, and analysis (Cloud). Likewise, Ref. [14] analyzed the procedures used to control Internet access and the confidentiality of IoT. The work presented challenges, opportunities in IoT security, and future directions. However, it did not use a systematic approach to investigate authentication and authorization methods for IoT. Confidentiality and security issues are examined in [15,16]. The limitations of IoT devices and corresponding security solutions are discussed. Moreover, a classification of IoT attacks and access control mechanisms is presented. However, it lacks discussion on IoT AA with the dynamic behavior of nodes.

In [17], the authors looked at IoT holistically in terms of device architecture, security, and privacy. They presented challenges for edge computing and IoT applications. In [18], the authors addressed the importance of the ML-driven methods for IoT security and privacy. In another study, Ref. [19] explored the viability of ML-driven schemes to identify intruders in IoT networks by applying these methods in intrusion detection systems, either by irregularities or traffic classification. Likewise, the authors [20] discussed the in-depth protection and privacy analysis of the layers (physical, network, and application). Moreover, the authors describe the shortcomings of the current ML-driven approaches and algorithms used for IoT security.

The author [7] presented a systematic review of the intrusion detection system (IDS) in IoT environments. A discussion on the advantages and disadvantages of the intrusion detection mechanisms based on anomaly, signature and other specification was provided. Detection accuracy, scalability, and IDS characteristics are also discussed in the paper.

Table 1 recapitulates the research contributions related to the authentication and authorization found in the literature.

### 1.4. Contribution

While several works exist in the literature, they are limited to specific IoT security and threats. A detailed survey of all existing and future security challenges, especially ML-based security schemes for authentication and authorization, is required for IoT applications. Our review will help the readers get a clear idea of the latest AA scheme for considering ML methods as an alternative solution to the current cryptography-based AA systems and provide an overview of the field. Specifically, our contributions are stated below:

- An overview of security and privacy issues with a taxonomy related to various authentication and authorization schemes in IoT.
- An overview of various types of attacks in each layer of IoT architecture leveraging a specific IoT AA mechanism.
- Recommendations for enhancing IoT authentication scheme for safe communications based on ML.
- An assessment of countermeasures of IoT security issues with ML for AA.
- Performance evaluation metrics for IoT AA for general and ML-based schemes are identified.
- Open issues, challenges, and future work recommendations for secure IoT system and services.

### 1.5. Article Organization

The rest of this article is organized as follows. Section 2 discusses the ML methods and Section 3 presents the taxonomy of AA for IoT, followed by IoT security requirements in Section 3.1. A layered view of attacks and risks in IoT for AA is presented in Section 3.2. Section 3.3.1 presents the general performance evaluation metrics and Section 3.3.2, presents the ML-based AA performance evaluation metrics. Section 3.4 presents recent AA techniques with their strengths and weaknesses. In Section 3.4.1, different IoT authentication schemes are described from the general point of view, such as encrypted schemes, and in Section 3.4.2, different ML-based IoT authentication schemes are described. In Section 3.4.3, different IoT authorization schemes are being described from the view of the attributes, roles and capabilities. Section 3.4.4 presents different ML-based IoT authorization schemes. AA characteristics are described in Section 3.5 based on the recent literature. In Section 4, the open issues, challenges, and future research direction are discussed. Finally, the conclusion is drawn in Section 5.

## 2. Machine Learning for IoT Security

In this section, we provide a brief overview of various machine learning algorithms and their use in IoT.

### 2.1. Supervised Learning

Supervised learning algorithms operate using labelled datasets and are used in IoT networks to detect spectrum, estimate the channel, adaptive filtration, and determine locations. This group includes two different process types: regression and classification. Support vector machine (SVM), naive Bayes, random forest, and decision tree are some of the most popular classification algorithms. Two widely employed regression methods are polynomial and logistic regression. Such algorithms are also known as “instance-based” algorithms that predict output based on the learned model for each new observation. Supervised learning algorithms, including SVM, DT, and naive Bayes (NB), have been widely used for IoT security. For instance, the non-linear constraints for a solution model are in SVMs. However, for massive data sets, SVM is inefficient. It is simpler to apply random forest algorithms and conform to the massive dataset available compared to SVM. It offers a greater degree of accuracy, which requires less time for prediction [21]. However, training requires more time than SVM and NB. Logistic regression and neighbouring algorithms require a lot of memory and processing of data with many features. Supervised learning methods have been used to detect intrusion, DDoS attacks for IoT networks in the communication layer and the cloud.

### 2.2. Unsupervised Learning

The unsupervised learning algorithms handle unlabelled data and heuristically use input data to learn patterns in the input data. In particular, unsupervised algorithms identify irregularities, patterns, anomalies, and cluster classes. Unsupervised learning makes use of classification algorithms to classify the data. In IoT, unsupervised approaches may be used without any knowledge of the desired output. Based on the unsupervised ML algorithms, two standard clustering algorithms are K-means and hierarchical clustering. Clustering using K-means is the most popular because it is a simple algorithm that forms clusters according to patterns detected in the data points (e.g., normal traffic or abnormal traffic). The cluster/edge groups are formed around the centroids, leading to the formation of same-size clusters. However, during the clusters’ formation, the number of clusters must be defined, which cannot always be done accurately and efficiently [22]. Unsupervised learning methods (i.e., K-means) are usually used to detect anomaly and Sybil attacks in the communication layer.

### 2.3. Reinforcement Learning

The reinforcement learning (RL) methods learn by exploring various actions in a given environment and find the optimal set of actions to maximize the reward. This reward arrangement is beneficial in overcoming various security issues in IoT [23]. RL requires no prior knowledge of the environment and learns based on experience by interacting with the environment to find the best action in a given state. RL approaches are simple, but determining optimal policy takes a long time. This slow convergence and an optimal state transition function or policy are the major issues in dynamic IoT network environments.

Reinforcement machine learning may respond to changes over time and adapt, as opposed to standard approaches such as linear aggregation. RL illustrates a significant aspect of developing an autonomous communications system that can efficiently secure IoT devices without external interference, intelligent enough to anticipate potential malfunctions. The reinforcement learning methods (i.e., Q-learning) can be used for IoT devices authentication, detecting jamming and malware attack by learning from the environment in the cloud or any high-computational edge devices without requiring a prior training dataset [24,25].

## 3. Taxonomy of Ml-Based AA for IoT

This section presents the proposed taxonomy for AA in IoT. The proposed taxonomy is based on the review of various research studies considering various aspects, while designing AA schemes for IoT security. Based on the analysis of existing literature, the research on AA can be categorized using the following classifications:

1. AA-based IoT Security Requirements
2. Attacks and Risks for AA in IoT
3. Performance Evaluation Metrics
4. Techniques used for AA
5. Characteristics of the AA schemes

**Figure 1:**
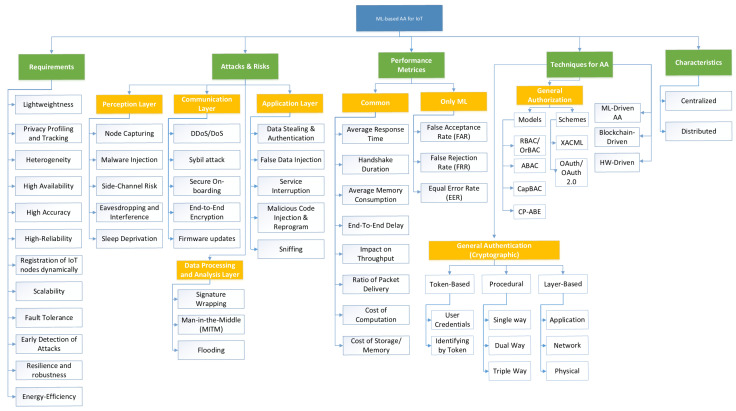
Taxonomy of ML-based AA for IoT.

Figure 1, illustrate the detailed taxonomy of ML-based AA for IoT.

### 3.1. AA-Based IoT Security Requirements

Due to the constrained nature of IoT devices and the heterogeneity, there are a wide array of requirements to secure IoT networks. IoT security requirements that need to be fulfilled for any AA systems are the following:

- **Lightweightness:** The first and foremost requirement is that the AA schemes must be required to be lightweight for IoT, such that, regardless of energy constraints, they work well for several IoT nodes [26,27,28].
- **Privacy Profiling and Tracking:** Combining an identification with a specific person is a vulnerability, which may escalate to privacy profiling and tracking [29]. Therefore, one of the critical problems is the dis-allowance of IoT operation, and the security system must be responsible for protecting the clients’ privacy.
- **Heterogeneity:** Within IoT, nodes and networks are heterogeneous. An essential requirement for the AA scheme must have heterogeneity because of end device characteristics. Heterogeneity in AA schemes will solve issues in multiple types of devices.
- **High Availability:** The high availability of the AA Scheme confirms that all network services are available to the greatest possible extent, even though threats are occurring within the IoT network [30].
- **High Accuracy:** A high degree of accuracy level of any AA scheme is required for accessing any IoT node and the network itself [31]. A high degree of accuracy level during authentication and authorization will ensure that the whole system is highly secure.
- **High Reliability:** One of the key requirements of any authentication and authorization schemes is high reliability. The method relies on its success rate. It confirms that all functions in the IoT network are operating correctly during the specified duration [32].
- **Registration of IoT Nodes Dynamically:** IoT requires networks with shorter life cycles than general computing networks, while IoT devices can dynamically be added and removed from systems [33].
- **Scalability:** A range of network scalability approaches is available to provide support to IoT devices, including the publish-subscribe protocols such as MQTT [34]. These scalability features should be combined with the security architecture.
- **Fault Tolerance:** Fault tolerance is the ability to continue working, even after one or more devices are compromised. If an end-device of the group such as edge node cannot authenticate with the group’s distributor such as cloud, the group loses its identity. This loss of identity causes the authentication failure of the other group devices. Therefore, suitable fault tolerant system architectures should be implemented [35].
- **Early Detection of Attacks:** The IoT network requires schemes and protocols after an attack is performed to guarantee that the attack is identified as quickly and efficiently before an attack does significant harm and spreads across the network. Moreover, the schemes are also needed to be tolerant to intrusions and various hostile attacks.
- **Resilience and Robustness:** Due to agility, IoT node faults and the rise in the number of attack vectors, the IoT AA system should be resilient to attacks and must be robust. Security networks should also be able to discover and repair faultiness by immediately taking the necessary steps.
- **Energy Efficiency:** Energy efficiency is an important metric to assess the lightweightness aspect of an AA system, as it is proportionate to the distance for the communicating nodes [36]. Besides that, lightweight programs with little code or less memory consumption are preferred in an AA system implementation.

### 3.2. Attacks and Risks for AA in IoT

Recently, AA in IoT has become a significant issue, as specified in the considerable amount of recent research works. AA is a critical issue for vendors in order to secure the IoT networks. Furthermore, the majority of IoT devices are expected to operate without human intervention. Therefore, it is required to have a method to authenticate and authorize devices and services without these human interactions.

**Figure 2:**
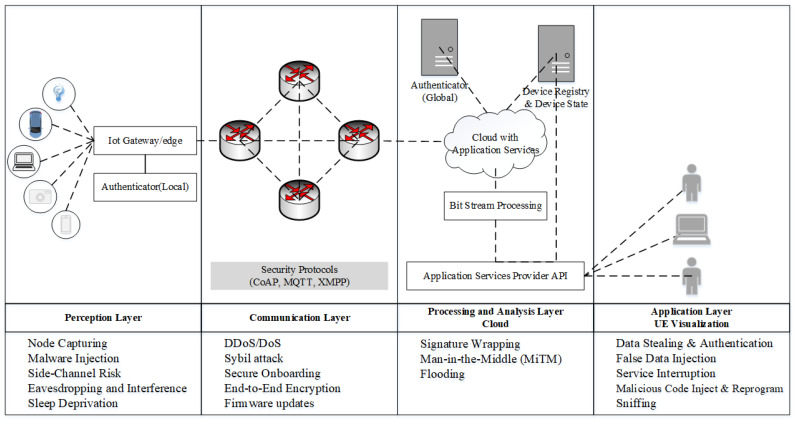
Layered view of IoT for AA and Security Risks.

The IoT networks can be divided into four important layers to simplify the development security mechanism. In the first layer, which is called the perception layer, specific sensors and actuators interpret data or knowledge and execute different functions. A coordination network for the transfer of aggregated data is included in the second layer called the communication layer. The data processing and analysis layer, known as the middleware layer, connects the network and the application layer in many evolving IoT applications. Finally, there is a range of end-to-end solutions in the fourth layer, the application layer, including smart grids, smart highways, smart industries, etc. All four layers have specific security problems. In addition to these, different gateways link these layers and help the transmission of information. These gateways also have certain security risks. Furthermore, each layer of the architecture of the IoT system has certain security requirements. Figure 2 described a possible architecture of IoT for authentication and authorization. The attacks for each layer for AA are discussed below based on IoT architecture.

#### 3.2.1. Perception Layer

The key objective of the perception layer is the detection of any event in the peripheral devices and the collection of real-world data. Such a layer comprises several sensors and can be called the sensing layer for the IoT architecture. One of the key advantages of IoT systems is that many sensors are used for applications. Sensors are typically embedded into IoT devices through sensor hubs. A sensor hub is a connecting point for many sensors that capture and reroute sensor data to the processing unit. For data flow between sensors and devices, a sensor centre uses various transport mechanisms, e.g., inter-integrated circuit (I2C) and serial peripheral interface (SPI). The transportation mechanisms rely on IoT devices and establish a channel of interaction between the sensors and the sensor data extraction applications. The security risks related to this layer are as follows:

- **Node Capturing:** IoT systems consist of many limited resource sensor and actuator nodes that are vulnerable to node capture attack. In such an attack, the attacker gains full control over the key node, such as the gateway. The attacker will then leak all information between sender and receiver, exposing the whole network [37].
- **Malware Injection:** The risks involve the injection of malware into the memory of the node by an external user. In general, IoT node firmware or software is modified to enable adversaries to insert malware to execute such unintended functions or even attempting to access the entire IoT network [38].
- **Side-Channel Risk:** In addition to direct node attack risks, separate side-channel risks may contribute to sensitive data leakage. These risks may depend on power use, laser attacks, time attacks, or electromagnetic attacks, which are also conceivable [39]. Modern chips take charge of numerous countermeasure steps in the cryptographic modules to avoid risks from this side channel.
- **Eavesdropping and Interference:** Many IoT systems consist of specific nodes distributed in open environments. An external party may secretly overhear information exchanged via different channels, such as a communication network, or authentication, in certain IoT applications, which might not be allowed [40].
- **Sleep Deprivation:** The attackers attempt to drain the battery from the low-powered IoT-edge devices during an attack. This can be accomplished by using infinite loops in the edge devices or deliberately increasing the device power consumption by using more power for data transmission [41].

#### 3.2.2. Communication Layer

The communication layer is a contact medium for transmitting data to other interconnected devices obtained in the perception layer. The communication layer allows data flow between devices within the same network using a wide range of communication technologies, such as Wi-Fi, Bluetooth, Zigbee, Z-Wave, LoRa, and mobile networks. This layer of an IoT architecture is also known as the network layer.

- **DDoS/DoS:** Many IoT devices in the IoT network can be used to launch DDoS attacks. This is used to exploit or breach the security of target servers. The Mirai botnet used this attack from numerous distributed nodes to flood the system by persistent transmission [4].
- **Sybil Attack:** This attack offers many separate identities for an individual node. For example, if there are many Sybil nodes in the same network, several requests from the legitimate nodes are rejected due to Sybil nodes in the same network [13]. Moreover, Sybil attack is possible from every layer of the IoT architecture.
- **Secure Onboarding:** In case of the installation of a new device in an IoT system, encryption keys must be protected. The gateways can be attacked and searched by the human being, especially during the embedded phase, to catch the encryption keys [42].
- **End-to-End Encryption:** To ensure the confidentiality of the data, end-to-end protection is required. For example, Zigbee and Zwave protocols allow encryption, but it is not end-to-end encryption. The gateways are needed to decrypt and re-encrypt messages to convert the details from one protocol to another [43]. This gateway stage decryption renders data prone to privacy infringement.
- **Firmware Updates:** Most IoT devices are resource-limited, so they are unable to access and install software updates with the user interface or computing resources [42]. In this circumstance, gateways are used to download and apply firmware updates by gaining access through authenticating and authorizing.

#### 3.2.3. Data Processing and Analysis Layer

The data processing and analysis layer comprises the data processing unit of IoT architecture. The collected data from the perception layer are processed and analyzed for deriving insights and making decisions. The data processing and analysis layer often preserves the output of the previous layer to enhance the security based on the device experiences. The outcomes from this layer are also able to be shared among the connected devices if necessary.

- **Signature Wrapping:** XML signatures are included in the cloud servers to maintain service integrity [44]. The intruder breaks the signature algorithm during a signature wrapping attack and may execute or change the eavesdropped information, utilizing the Simple Object Access Protocol (SOAP) vulnerabilities.
- **Man-in-the-Middle (MiTM):** The MQTT protocol employs the MQTT Broker as a publishing subscriber communication model for customers and effectively acts as a proxy. In MITM, the attacker may gain control of the MQTT broker by becoming an intermediary node in the middle and trying to tap the information of other nodes without knowing the potential client nodes [13]. Similarly, many IoT message queueing protocols, such as SMQTT, AMQP, CoAP, M3DA, and XMPP [45], can be exploited using MITM attack.
- **Flooding:** This flooding is fundamentally the same as the cloud DoS attack, impacting service efficiency (QoS) by submitting numerous queries [13]. The strain on the cloud servers will have a significant effect on cloud-based systems.

#### 3.2.4. Application Layer

In the cloud application layer, the data processing layer schemes are implemented to achieve different IoT device security levels. The IoT device application layer is a user-centred layer that handles specific user tasks. Possible IoT application layer attacks are:

- **Data Stealing:** Many sensitive and private details are protected by IoT applications. Transit data are even more vulnerable to attacks than data at rest. A lot of data movement occurs in IoT applications. When such systems are vulnerable to data stealing threats, individuals would be hesitant to disclose their private data in IoT systems [46].
- **False Data Injection:** If the node has been captured, the intruder can insert erroneous information into the IoT application that can generate false results and cause IoT application to fail. DDoS attacks may use this critical approach too [47].
- **Service Interruption:** Such threats are often classified in current literature as unauthorized disruption activities or DDoS attacks. There have been many cases in which IoT systems have been targeted. These threats temporarily blocked legitimate customers from accessing services on IoT infrastructure by deliberately exploiting servers or the network.
- **Malicious Code Injection and Reprogram:** Attackers are typically used to hacking through an IoT device or network utilizing the fastest or shortest route. An effective XSS attack can cause the device to hijack an IoT account and paralyze the IoT system if it is prone to malicious scripts. Still, the attackers may try to reprogram IoT objects remotely using flaws in the logic of the program [48].
- **Sniffing:** To access sensitive user data, an intruder can use a sniffer system to monitor network traffic in IoT applications when protection protocols are not enough to prevent it [49].

### 3.3. Evaluation Metrics for Authentication and Authorization (AA)

In this section, we discuss commonly used metrics to evaluate the various AA schemes.

#### 3.3.1. General AA Evaluation Metric

1. **Average Response Time:** The time required to reply to the client request is the response time for a server node or gateway node (GWN). Several variables influence this, such as server architecture, the number of clients, the bandwidth of the network, volume of requests, request types and calculation time. Equation (1) can be used to measure first response time.
  (1) $$Time_{res}=time_{fir-res}-time_{req}$$
  Here, $Time_{res}$ indicates the total time taken for the response, $time_{fir-res}$ indicates the first response time, and $time_{req}$ indicates the client’s time of request. The average response time will be determined by the mean of all response times, as seen in Equation (2).
  (2) $$Time_{avg-res}=\frac{n_{user}}{req}-Time_{think}$$
  where the average response time is $Time_{avg-res}$, the number of simultaneous users is denoted by $n_{user}$. The volume of requests that a server gets is req. $Time_{think}$ is the average time of thought (in milliseconds). It is necessary to use think time in the equation to achieve exact response time.
2. **Handshake Duration:** Handshaking is a negotiating mechanism between two parties (e.g., client, actuator, sensor node, server or other nodes) in an IoT network. Handshaking occurs by finishing a two-round trip message. The server identification of the client and the server request recognize [50]. The handshake duration can be denoted by $Time_{handshake}$ and shall be calculated by Equation (3) at the client-end.
  (3) $$Time_{handshake}=Time_{session}+Time_{res}+Time_{processing}$$
  where the time taken from a complete session request is denoted by $Time_{session}$, the response time of the client is indicated by $Time_{res}$ and server processing time denoted as $Time_{processing}$. Random handshakes between the client node and server node are performed to determine the duration of the handshake. Based on the handshake duration, the standard deviation is calculated to observe the variability.
3. **Average Memory Consumption:** IoT is a network of primarily wireless sensors, which are constrained in memory and other resources. Therefore, in specialised and autonomous sensor networks, memory usage optimization is critical [51]. The usage of memory varies at different levels in IoT applications, including the user, sensor, gateway, etc.
4. **End-to-End Delay:** The average time for delivering packets from sending node to the receiving node is defined as an end-to-end delay and denoted by E2ED. Using Equation (4), the E2ED can be computed.
  (4) $$E2ED=\frac{\sum_{k=1}^{n_{received-pkt}}\left(Timestamp_{i}^{r}-Timestamp_{i}^{sent}\right)}{n_{rcvd-pkt}}$$
  Here, packet number is denoted as *i* and the number of received packets is denoted as $n_{rcvd-pkt}$. Similarly, $Timestamp_{i}^{rcvd}$ denotes the received timestamp and $Timstampt_{i}^{sent}$ denotes the sent timestamp for *i*-th packet. E2ED is directly proportional to the number of nodes in an IoT network.
5. **Impact on Throughput:** The throughput can be defined as the amount of data passing in a unit of time through a node. In [36], the authors demonstrated that the output could be represented by Equation (5).
  (5) $$IMPACT_{TP}=\frac{\sum (Qnty_{i}^{rcvd}\times lngth_{i})}{Time_{whole}}$$
  Here, the impact on throughput is denoted as $IMPACT_{TP}$, while $Qnty_{i}^{rcvd}$ denotes the received quantity and $lngth_{i}$ denotes the length of the *i*-th packet, and $Time_{whole}$ denotes the duration to complete the simulation.
6. **Ratio of Packet Delivery:** Packet delivery ratio denotes the number of packets transmitted by the sending node and the number of packets successfully received at the receiving node. It depends on several variables, such as network setup, system capacity and bandwidth. Equation (6) can be used in the calculation of the packet delivery ratio.
  (6) $$RATIO_{PD}=\frac{Number_{pkt-rcvd}}{Number_{pkt-sent}}$$
  where $RATIO_{PD}$ is the packet delivery ratio. The total number sent by the sending node is denoted as $Number_{pkt-sent}$ and the number of packets received by the receiving node is $Number_{pkt-rcvd}$. When a message is transmitted through the networks, energy is consumed. When more packets are transferred across the nodes, the energy consumption will increase accordingly [36]. So, if the energy level is below a threshold or reaches the node constraints, the packet can be discarded at the edge or cloud.
7. **Cost of Communication:** As stated in the literature, the number of interactions during authentication will differ and depends on the protocols in use. Moreover, it will require different phases and a specific number of messages to establish communication. Furthermore, the scheme involved in the AA process required at least four messages to complete authentication, such as messages transfer between the sensor, GWN, and the user or the central node to establish a secure communication through authentication [36,52,53,54,55,56]. Therefore, the number of messages varies, and different communicating messages carry different information. Due to this, there must be a consideration for communication costs, since multiple standards are subject to varying threshold values. For example, the networking protocol of IEEE 802.15.4 accepts 127 bytes. In comparison, the standards IEEE 802.15.6 carry a limit of 255 bytes of frame length.
8. **Cost of Computation:** Computation also relies on protocols within the IoT network. As most network devices are computationally limited, computing large data or information of IoT networks is not feasible. Therefore, protocol architects aim to build lightweight IoT network authentication protocols. In this regard, the principle of hash, XOR, and concatenation has been used by researchers to protect the message across the network [51,57]. In IoT, authentication schemes, such as ECC-based and Fuzzy extractor, are also enforced for authenticating the identity of IoT devices [58].
9. **Cost of Storage/Memory:** Protocols employ various kinds of schemes to implement IoT authentication. One of the common strategies is a smart card. A smart card can store some data, such as sensor information, login credentials, and gateway node information [59,60]. To accomplish authentication, multiple protocols use various functions for improving efficiency, and among them, one of the significant performance metrics is the cost of storage/memory.

#### 3.3.2. ML-Based AA Evaluation Metric

There are several performance metrics used to evaluate ML-based AA schemes, including the metrics mentioned above. The following metrics can be used to evaluate ML algorithms for enhancing the performance of ML-based AA schemes.

1. **False Acceptance Rate (FAR):** The ML-based AA technique may incorrectly recognize the characteristic of deceivers based on the classification threshold preference. The FAR is the threshold, which depends on falsely accepted characteristics, divided by the number of all deceiver characteristics. It is also known as the false alarm rate. The value will be between 0 and 1. When all the false characteristics are accepted as valid, then the value will be “1”, and the value will be “0” if none of the wrong features are considered valid [61]. Equation (7) presents the formula to calculate FAR.
  (7) $$FAR=\frac{N_{FA}}{N}$$
  where $N_{FA}$ is the number of false acceptance cases and *N* is the number of attempts.
2. **False Rejection Rate (FRR):** If an overly high classification criterion is used for the ratings, certain trends for individual characteristics are wrongly denied. Depending on the threshold value, none or all characteristics are denied falsely. The false rejection rate (FRR) is the number of rejected characteristics divided by the total number of individual characteristics [62,63]. Equation (8) presents the formula to calculate FAR.
  (8) $$FAR=\frac{N_{FR}}{N}$$
  where $N_{FR}$ is the number of false rejection cases and *N* is the number of attempts.
3. **Equal Error Rate (EER):** When the distribution of the similarity and dissimilarity value of a pattern converges, FAR and FRR overlap. Picking an accurate threshold value becomes an issue. The EER is named at this point, which will be the same for all other characteristics [61,64].
4. **False/True Positive:** An actual result under which the model accurately forecasts the positive feature vector is truly positive. Likewise, an actual result in which the model wrongly forecasts the positive feature vector is a false positive [24].
5. **False/True Negative::** An actual result under which the algorithm forecasts the negative feature vector accurately is a true negative. On the other hand, false negative is a concept in which the negative feature vector is falsely predicted as an outcome [24,64].

### 3.4. Techniques for AA

Authentication is a crucial prerequisite for IoT security. For accessing any IoT applications or entities, the receiver node must authenticate the participants. IoT applications and utilities are typically focused on data interactions through different networks. The data obtained from IoT systems are analyzed and then transferred through a decision-making mechanism. Such mechanisms may differ based on IoT design, and the data flow in such applications may be similar. For example, an application or a user needs some data from an IoT device without loss of generality. In that case, The IoT device should authenticate the sender node to the IoT network. The requester should be guaranteed access rights for the data or the node required. The request for accessing such data or node is otherwise refused. AA’s is of utmost importance in IoT networks due to their roles in controlling the trust management level in the network and in preventing impostors claiming to be legitimate IoT devices. Additionally, the exposure of sensitive information to IoT applications or nodes must be revoked for all users after a specific inactivity duration. In this section, we discuss various authentication mechanisms. Before discussing ML-based AA schemes, we present various categories of AA found in the literature.

#### 3.4.1. General IoT Authentication Schemes

IoT authentication security aims to identify and remove malicious nodes from the network [5,65]. The authentication process is a primary phase to validate the identity of the participating nodes in the IoT network. Even though only a few are considered to be ML-based, most studies in the literature emphasize detecting malicious nodes. Due to the scalability issue and the large number of things involved in exchanging sensitive data, intelligent AA security management is needed to enable multiple authorization factors to specific nodes.

Device-based authentication and authorization use client-side certificates stored on the machine. Verification occurs during the TLS handshake. As part of the handshake, the device sends its certificate, signed by a pre-configured signing authority. It sends the signed information with its private key. At the end of the handshake, the device is verified, and the client ID is removed from the certificate. For a machine, the customer ID may be the machine identification number (MIN) which is vital for verification from the server.

Once the machine has been verified, the customer ID is known. The next step is to determine the correct authorization groups. When connecting, a device can be part of a permission group. For example, a device is a machine, so all connecting machines are given the “machine” authorization group. It is possible to extract additional groups from the certificate and client ID. For example, it is possible to extract different groups of MIN from the machine. The mutual authentication of IoT systems is introduced in [66], which is established for client–server communication using the lightweight Application Layer Protocol CoAP (Constrained Application Protocol). Advanced encryption standard (AES) benefits are used as a secure communication channel. Authentication takes place on client and server challenges by encrypting the maximum payload size and interacting with the control loads. Authentication is performed during a messaging interaction without using an additional layer (DTLS), which increases communication and computing costs. The author also considered the robustness of the authentication scheme and dynamic registration of IoT nodes. Furthermore, average response time, handshake duration, and average memory consumption are discussed to benchmark the performance of the scheme. Additionally, the author showed attack tolerance to combat DoS-type attacks. The authors have not considered privacy and have not discussed the pre-shared key at the provisioning stage.

In [67], researchers proposed a lightweight multi-factor authentication strategy. The researchers utilized digital signature and device capacity for multi-factor authentication. The device capacity is similar to the resolved computational functionality of the device, which can be a mathematical problem-solving challenge or even a primitive puzzle based on cryptography. Moreover, authentication can utilize a strategy to authenticate both the end node and the server node. It can ensure security for both the application layer and physical layer. The end node sends a transmission request to the server through a protected TLS channel. The server node then transmits a private key and an encrypted timestamp message, which would prevent repetitive attack attempts. The end node decrypts the signature, addresses the ciphertext by functional procedure, registers the result with its personal key and returns them to the server node. The researchers also have explored MITM attacks and replay attacks tolerance. Eventually, the server node validates the signatures and the outcomes from the function during device authentication.

In [68], an Internet of Medical Things (IMT) authentication is proposed for devices that use human physical capabilities to communicate securely. Biometric techniques have been used for environments, while the analysis shows compatibility and safety, especially smart health. The authors presented the requirements for an intelligent health environment and discussed some of the open problems and challenges of biometric authentication.

A new authentication scheme for IoT systems has been proposed Bubbles-of-Trust by the authors of [69] which is based on blockchain. This mechanism aims to create bubbles by dividing devices into virtual zones to identify and trust each other through grouping. An Ethereum public blockchain is then applied to control and approve the transaction between devices. However, to validate a transaction, this consensus protocol takes a considerable amount of time, which is not feasible for real-time applications. Moreover, the transaction fee in the public blockchain is considered inefficient.

In [70], the lightweight two-factor authentication scheme is proposed to enable the authentication of Internet systems integrated with the one-way hashing and XOR-ing in the cloud computing environment. Registering, verifying, and updating the password are steps in the authentication process. Moreover, costs and computational efficiency are also considered and demonstrated in an environment where resources are minimal. However, the cost of communication and the computation cost at the cloud are also higher and have not considered the attacks, e.g., DoS and DDoS.

In [71], researchers proposed a rigorous assessment of WSN-supported IoT trust models focused on various elements, such as interoperability and resource optimization. The analysis found that neither of these models had (1) attempted to bring together data fusion trust (DFT), communication trust (CT), and data perception trust (DPT) and (2) met IoT security requirements. Therefore, it is essential to provide integrated trust protection to the platform. This model aims to evolve the resources as per IoT applications authentication requirements.

In [72], the researchers proposed a method to manage the firewall rule that takes all firewalls on the network route into account. As every network firewall is implemented, anomalies between rules of the various firewalls may arise. The authors of [73] discover and correct these erroneous configurations using a formal data structure method. The process also allows optimization of simple firewall rule sets by elimination rules which are no required anymore. Although the initial objectives are to detect the wrong internal firewall configurations, it did not focus on resolving incorrect configurations in a distributed environment during the analysis and verification of other network components security configurations. The researchers of [74] identified attack patterns and ML for IoT security techniques, including IoT user authentication, malware detection, role-based access control, and secure downloading. It addresses several challenges in implementing security techniques for real IoT systems using ML, such as partial observation of the states, computational overhead and communication overhead, and backup security solutions.

Although only 20% of access control methods currently use trust assessments, according to the researchers of [75], it is still a promising security mechanism because of its ability to calculate node dynamic trust value. The process helps the trust rating of each node to be measured gradually. Moreover, the authors of [76] proposed using ML as an intelligent trust assessment to mitigate an on-off attack during authentication that would threaten the node’s trust value. Additionally, security protection should introduce apparent authentication vulnerability, such as compromised node attacks. The researchers claim in [77] that TBAC (trust-based access control) reliable access-control computing on IoT networks is still relatively recent but widely deployed in commercial applications. The authors of [78] also suggested a control scheme that supports multidimensional reliability properties utilizing trust and IoT reputations. The trust assessment is centralized just as in other work owing to the resource constraints of the devices.

In [79], the researchers used PKI benefits by using X.509 digital certificates To ensure secure authentication among devices within IoT systems. For device identification and device integrity, such a certificate can be used. But analysis of security and the requirements of scalability for PKI-based IoT systems are not considered. The authors of [80] implied a new easy authentication protocol that uses physically unclonable function (PUF)-based RFID tags, where the steps consist of tag recognition, validation, and updating. Mostly, the tag is recognized by the reader. Second, the reader and the label mutually verify authenticity. Finally, the most recently used password is updated for the next confirmation. However, the authors have not considered it in various environments.

Datagram Transport Layer Security (DTLS) protocol allows the client-server application to communicate securely over Transport Layer Security (TLS) protocol [81], while TLS deals with the information forging, altering, and fragmentation [82]. DTLS deals with re-ordering packets, loss, and size of the datagram without considering Denial of Service (DoS) attacks. Authors in [50] proposed Enhanced Lightweight DTLS for the Internet of Things (E-Lithe). The communication starts with sharing a pre-shared key between the server and the trusted third party (TTP). In the next step, the TTP shared a mutual key with the client, which allows the client to communicate securely with the server. After this phase, a handshake request from the client takes place with the server. The server validates the mutual authentication key. In the last phase of authentication, the server transmits a Hello message to continue communication securely if the key matches. Otherwise, the communication has been terminated by the server for further communication. The E-Lithe scheme uses NHC (next header compression) and IPHC (IP header compression) as compression schemes. Despite the benefits of decreasing DoS attacks and computational overhead compared to other Lithe and DTLS methods, the multiple create requests and multiple handshake requests from multiple requesters can cause drainage problems as it needs to control numerous computations.

In [83], the researchers proposed a hardware-driven authentication scheme for classical RFID tags for anonymous authentication of RFID systems. They then provided an improved scheme for a noisy physical unclonable function environment. The main disadvantage of this scheme is that it did not consider the challenge-response pair (CRP) server comments when an existing group is empty. New mutual authentication and key management for WSN systems based on a biometric and symmetric cryptographic system in presented in [84]. The authors used BAN logic for the exactness of the mutual authentication and AVISPA tools to validate computational and communication costs. Moreover, the security risks, requirements, and functional attributes are compared with the other system considered in the literature.

Table 2 represents the benefits and limitations of the selected authentication schemes and Table 3 summarizes the recent studies on authentication model obtained in the literature. Moreover, Table 4 summarizes the performance metrics and attacks considered in the literature. It can be observed that all the authentication schemes for IoT are implemented across the layers are not hybrid and triple-way procedural. However, less research has been done for the single-way procedural method, while most authentication schemes focused only on the encryption-based schemes.

#### 3.4.2. Ml Based Authentication Schemes

Available security techniques cannot be used in the IoT physical (PHY)-layer due to the resource and scalability constraint. Intelligent AA schemes must be flexible and robust enough to manage the large number of nodes involved in the IoT network. The techniques for PHY-layer authentication using spatial correlation of wireless channel characteristics, such as received signal strength indicators (RSSI), channel impulsion response (CIR), channel state information (CSI), and media access control (MAC) address, can provide lightweight security mechanism for IoT devices. However, due to the dynamic nature of the wireless channel, the first three parameters are characterized by spatial and temporal distributions, making them hard to track. Intelligent ML-based AAs can continuously learn and validate the ever-changing PHY-identity of the wireless node. For example, in [62], hypotheses tests to equate the PHY-layer characteristics of the received data under evaluation with the transmitted data is presented. The performance of authentication depends on the threshold value of the hypothesis analysis. However, it is challenging for an IoT system to select an acceptable threshold for the authentication evaluation model because of the wireless environment and the uncertain spoofing structure. IoT systems may often use RL techniques to determine major authentication parameters without being aware of the network layer model by treating authentication like the Markov decision process (MDP). Usually, authorization for IoT systems has been complex in heterogeneous networks with a large number of nodes and multi-source data. The authors in [85] discussed intrusion detection by different ML techniques such as SVM, K-NN, and neural networks. In [86], the authors proposed a multi-variate analysis of correlations to extract geometric correlations between network traffic elements for DoS attack detection. In contrast to the closest triangular-zone neighbour solution utilizing the KDDCup99 datasets [87], this scheme raises the detection accuracy by 3%.

In [62], the authors proposed a Q-learning authentication model with a constant threshold by considering the RSSI signals. The proposed method allows an IoT device to achieve an optimum experimental threshold and improve the utility and the authentication accuracy. However, IoT nodes such as outdoor sensors have limited resources and computational limitations, which usually degrades IoT system performance for network security anomalies. ML algorithms aim to develop lightweight authorization protocols to save resources and improve IoT system lifespan. In [88], the authors developed a deviational detection system based on a K-NN to address the unmonitored deviation detection problem in wireless sensor networks and provide flexibility with reduced power consumption. In comparison to the centralized frameworks, the proposed framework results in 61.4% of energy.

In [61], the authors demonstrated better resistance to spoofing in IoT systems using supervised ML techniques like DFW (Frank–Wolfe) and IAG (incremental aggregated gradient). To simulate the scenario, the authors considered multiple sites, and each site consists of multiple antennas. The RSSI received from multiple site marks utilized to reduce the overall overhead of communication, improve the spoofing detection accuracy and the average error rate of IAG authentication. The DFW saves communication overhead by 37.4%, while the IAG saves the computational overhead by 71.3% compared to the other authentication schemes.

In [89], the authors implemented an unsupervised learning authentication approach for nearby IoT devices without leaking location information from their devices. The technique is based on a Bayesian non-parametric procedure IGMM (infinite Gaussian mixture model) to calculate the RSSI and the duration between packets to identify spoofers beyond the vicinity of low-frequency wireless signals. The proposed method improves the rate of error detection in spoofing tests on Euclidean distance-based indoor authentication. This scheme requires IoT devices to transmit the low-frequency signal features such as RSSI, time of arrival of a packet, and MAC addresses internally to the low-frequency signals received during a particular time frame. The nodes extract and transmit the low-frequency signal characteristics to the legitimate recipient. When such authentication responses are received, the recipient applies IGMM to start comparing the signal elements reported with the actual signal features identified in the proximity-based experiments. The recipient replies to IoT nodes with connectivity to IoT resources when authenticated.

IoT offloading must address PHY or MAC layer attacks such as jamming, fake edge nodes, fake IoT nodes, eavesdropping, man-in-the-middle attacks, and smart attacks before deploying in any environment is necessary. Since the current status and offloading policy in an IoT system is independent of the previous status and actions, the offloading strategy for portable devices used by IoT nodes in repetitive games with interceptors and interference sources can be perceived as a finite status. In diverse wireless settings, AA may use RL strategies for the optimization of updating procedures. The node analyzes the signal to interference plus noise ratio (SINR) of the frequencies received, the secrecy capability, the residual offloading, and the offloading technique’s energy usage and forecasts the usefulness. IoT node uses the greedy mechanism to select the offloading strategy to maximize Q-function and other policies, thereby ensuring reconciliation between exploration and exploitation. This procedure limits the risk of spoofing and decreases the impact of jamming relative benchmark as illustrated in [25]. The authors of [25] also illustrate a model-free RL Q-learning technique for offloading, which is easy to implement and requires less computational resources. It is also possible to choose its offloading throughput to avoid jamming and spoofing attacks. With this approach, the IoT device estimates the significance of the job, the obtained jamming force, the maximum throughput of the wireless channel, the channel gain from the channel to construct its current status, and the necessity for choosing a Q-function offloading strategy. The Q-function is the assumed long-term discount rate reward for every execution-state pair and the previous offloading information against jamming. The Q-function is modified in each time slot through the iterative Bellman function to the current offloading strategy, the network status, and the jamming tool obtained by IoT nodes.

In [90], the authors proposed a Q-learning-based anti-jamming approach, where a node can choose a wireless channel to access the cloud or edge device without knowing IoT systems interference or jamming framework. IoT node monitors the center frequency and wireless bandwidth of each channel. It selects the optimal offload channel according to its current state, Q-function and perceived state. When the IoT node receives the computational assessment, the usefulness is calculated, and the Q-function values are modified. The average cumulative reward is higher than the other random channel selection strategy. The Q-learning method also allows IoT systems to achieve the best wireless spectrum frequency sub-band for jamming and other network equipment disturbance. As shown in [91], the IoT device monitors the spectrum occupancy to represent the status and selects it as a selection policy.

In [92], the authors proposed a smart user authentication technique illustrated by the simulation result that the enhancement of authentication accuracy using deep neural networks (DNN). The main limitation of such kind of application requires more computational and memory resources. The process starts with extracting CSI features from the requester’s signals and then uses DNN to detect spoofing type attacks. Furthermore, the DNN-based spoofing detection scheme accuracy is above 94.5%, while the accuracy for user identification is approximately 93%. The authors of [93] proposed a DQN based two-dimensional anti-jamming transmission, which would allow the specified frequency channel to be selected, speed of learning for IoT devices with sufficient calculation and memory resources. This scheme uses the convolution neural network to suppress the space in a dynamic IoT system with large-scale networks, thus enhancing the SINRs of received signals with many IoT nodes and jamming strategies. However, in contrast to the Q-Learning method for offloading across jamming, this system saves more than half of the time. In [94], the authors proposed a Q-learning game-theoretic method to detect DoS attacks in IoT using SINR. The outcome shows that the learning and detection accuracy against the powerful attacker are higher.

IoT nodes may use supervised ML techniques such as KNN to evaluate runtime behaviours for malware detection. As defined by [88], and IoT node uses KNN and random forest classification tools to create malware features extractor. The IoT devices filter the TCP packets, select features from a number and length of the frame, and store them in the database, among various networking features. IoT nodes may also move code monitors to a protected server or edge systems for malware perception with repositories of software, higher processing rates, increased memory, and better protection services. But the authors [24] have suggested RL techniques that can be used to ensure the optimum offloading strategy for an IoT node without knowing about the malware framework and app generation framework in the dynamic malware detection game. It makes use of Q-learning for the optimum offloading rate for mobile devices without understanding the trace generation of the adjacent IoT nodes and their wireless bandwidth models. IoT nodes divide the environment into several zones in real-time and monitor the user density and the bandwidth of the wireless channels to formulate the existing state. In this time frame, the IoT node estimates the precision benefit, latency detection, and energy consumption of the utility. The authors proposed the Dyna-Q malware identification method in [24] that uses the Dyna framework to learn and identify the best offload approach. To improve learning performances, both the real experience and the simulated experiences generated by Dyna architecture are used.

Table 5 summarizes the benefits and limitations of different ML algorithms used in the literature along with their performance accuracy and Table 6 summarizes the recent research on ML-driven authentication schemes obtained from the literature. There are no standardized performance metrics used as they vary across different layers. Furthermore, none of the proposed schemes addresses mitigating all types of attacks, indicating the difficulty in introducing robust design across different attack models. Time taken for authentication is one of the most important performance parameters which is not considered in the literature. Furthermore, RL is one of the most promising algorithms to solve the listed AA attacks in different layers obtained from the literature.

#### 3.4.3. General IoT Authorization Schemes

IoT authorization schemes are dependent on node characteristics such as attribute, role, and capability. Access rules can be specified by different attributes, such as topic characteristics, object characteristics, environment characteristics within the attribute-based access control (ABAC). Static components, including users, responsibilities, passwords, requirements, and devices, are used in the role-based access control (RBAC). There are several interactions between these components. Mining the relationship is also one of RBAC’s key activities. Capability-based access control (CapBAC) always creates a lookup table to search for capabilities in functions and resources permission of every domain, such as a token and key. The exact individual has limited access privileges in different domains. Therefore, the physical aspects of the products, such as biometrics or system product ID become the main focus. However, the RBAC and ABAC authorization schemes do not provide the benefits for the distributed networks with multiple interactions, complexity, and dynamic scalability of IoT specifications, with adequate management and effectivity. A similar challenge to access control lists (ACL), the concept of least privilege is challenging to implement in RBAC and ABAC systems.

1. **Attribute-Based Model** In the ABAC-based model, the object and the subject are both defined by the attributes function [96]. The client is given a suitable right of access in the ABAC model in compliance with his characteristics. Recent studies mainly concentrated on maintaining user privacy because attributes could include user private information whose leaking significantly hinders ABAC expansion. In [97] the authors introduced a privacy-preserving attribute-based access control (P-ABAC) scheme to avoid data leakage. In P-ABAC user-side manages the critical attributes with homomorphic encoding. With the assistance of stable ciphertext-based homomorphic multi-party computational encoding techniques, the authorization mechanism can also make error-free decisions about access by the received attribute without any knowledge about privacy.
2. **Role-Based Model** The role-based access control (RBAC) offers system permissions that stipulate access for the users to services depending on individual roles and encourages security principles, such as task separation, administrative segregation of administrative roles, and fewer privileges. When the tools extend or access rules span multiple administrative realms, the RBAC model alone has a function eruption dilemma. A service-based RBAC model will be able to run in more IoT scenarios. Moreover, an expanded RBAC model using contextual knowledge, which can be used in detail as constraints to achieve a more scalable, modular, and lightweight access control mechanism. In [98], Barka et al. recommended that RBAC be implemented in smart entities across the Internet as a Web of Things (WoTs) access control mechanism.
  The organizational role-based access control (ORBAC) model is an extension of the RBAC model. It introduces a new dimension to the concept of “organization”. In the trust-ORBAC model, the ORBAC model uses the concept of trust management. Trust-ORBAC defines two dynamic thrust vectors, one for the organization and the other for users with various parameters. Moreover, the Tr-ORBAC model in which trust is embedded to improve the cooperation of various organizations and avoid malicious behaviour. In the SmartOrBAC scheme, the problem is grouped into smaller functional layers. SmartOrBAC distributes the processing costs between restricted and less restricted equipment to ensure that some users can access data in advance in the context of the task/action plan by restricting access to confidential information beyond the control of the system administrator. In [99], Guesmia et al. suggested an expansion of the OrBAC paradigm with a non-monotonic logic of the temporal representation. This logic may be a centralized plan to reflect the policies formally. The schedule contains a variety of assignments ordered, and in specific situations, make exceptions. The proposed OrBAC model can dynamically evaluate the required action sequence according to the data environment patterns while the access request is generated.
  In [100], the authors proposed a framework of access control and verification schemes based on location-controlled functions named location-constrained roles-based access control (LCRBAC). The authors describe cyberspace and physical space as a static topology for the LCRBAC scheme by describing cyber objects and physical object dynamic behaviors. The proposed scheme is often focused on the verification findings on a labeled transfer device to allow cyber-physical experiences via the reaction policy and principle.
3. **Capability Based Model** In capability-based access control (CapBAC), users provide access through an authorization sequence and token (such as authorization key, service ticket). Identity management is not a key function that offers tremendous benefits, especially where clients can access interdisciplinary scenarios. The comparison between ACL and CapBAC is that access control depends on the central server in the ACL model. On the other hand, the CapBAC model has leaves access management responsibility to the client. The BlendCAC concept, which is a decentralized CapBAC blockchain operating model, was proposed by Ronghua Xu [101]. A clear identity-based token management approach for access control, which uses a clever contract to register, spread, and revoke the authorization, is proposed within the BlendCAC model. In a cloud-based authentication scheme, users can access IoT services and command multiple smart ubiquitous environments through IoT cloud. In the case of large IoT schemes, Xu et al. [102] introduced the Federated CapBAC Mechanism (FedCAC), which is a token management approach focused on the identification that requires registration, dissemination, and revocation of the entry.

#### 3.4.4. Ml-Based Authorization Schemes

For several research conflicts and resolutions, policy developers are expected to contribute to the policy’s arbitrary and versatile modification manually. Furthermore, there is no successful method for identifying conflicts of interest and managing policy for IoT services authorization in the current world of coexisting security conflicts. Researchers are attempting to use ML-based techniques to build semi-automated authorization schemes. In [103], the authors formulate the mutual intrusion detection system as an evolutionary game-theory model. The model offers a solution to help nodes determine whether or not to take part in the detection. The intrusion detection scheme will resolve issues in which mutual nodes do not fully know other nodes.

In [104], the authors represent a scheme for resolving disputes between IoT-based services by redefining conflict resolution as a planning problem. They establish a semantic policy structure for IoT-based conflict identification and allow soft constraints to settle conflicts resulting from multiple services. In [105], the authors perform a detailed survey of current frameworks and systems of access management applied to shared community-centered systems. As the authors suggested, authorization schemes should promote easy-to-use semi-automated dispute resolution procedures and cohesively permit straightforward data processing in the event of contradictions rather than implementing blanket policies. The authors in [106] implemented authorization schemes based on the multi-layer perceptron (MLP). It uses the two neural networks on the hidden layer to train the MLP connection weights and compute the suspicion that an IoT device suffers from DoS attacks. This scheme utilizes back-propagation (BP) for the retrospective estimation and error BP and particle swarm optimization (PSO) as a reproductive adaptation for measuring the link weights of the MLP using particles with variable frequencies. IoT device is tested to shut down the MAC layer and the PHY layer for the conservation of energy and increase network life by exceeding the threshold for the output of MLP.

### 3.5. Characteristics of IoT AA Schemes

#### 3.5.1. Centralized AA

The central entity is often referred to as a trusted third party in centralized AA schemes. It is involved in interaction that collects, stores, computes, monitors, and distributes each network entity authentication factor (value). The PKI-based SSL/TLS is among the most frequently used authentication scheme using a centralized CA. While PKI has several trustworthy certificate authorities (CA), the AA scheme is still centralized and can cause a bottleneck in a highly dynamic and heterogeneous IoT environment.

One of the predecessors of IoT, the wireless sensor network (WSNs), has its own security services. Many WSNs use a comprehensive network central server to manage sensor nodes operated by low-powered batteries. A gateway/edge node also acts as a root of trust for perception nodes, specifically as a large distribution unit. Sensor Network Encryption Protocol (SNEP) is an example of Security Protocols for Sensor Networks (SPINS) in a typical case. Throughout SNEP, any node with its edge node shares a hidden key named the master key. Additional keys are extracted from a unified authority master key between perception nodes.

In [107], the authors proposed a cross-domain and locally organized robust distributed trust management (RobustTrust) authentication system. The trust has 3 security components that assist with the use of the expert knowledge-based framework to enhance security system efficiency by evaluating trust on every node to combat compromised and malicious devices/nodes. In addition, the node may share the knowledge of the distributed trust assessment with one specific node. The trust parameter evaluation consists of the acquisition of information about the other nodes (based on compatibility, integrity, and response), credibility assessment (based on honesty, consistency, and cooperativeness) and node interaction (based on competency, suggestion, and trustworthiness). However, node vulnerability and the trust management strategy in the cross-domain environment are not assessed in terms of security.

In [108], the researchers proposed a holistic cross-domain trust management model (HoliTrust). It is a multilevel and centrally managed security concept for authorization that considers cross-domain node-to-node communication aspects and cross-domain trust management significance neglected in previous studies. The HoliTrust system was developed by segregating domains into communities where each community has its dedicated server to calculate trust level, based on the similarities and interests of the request to provide security to communities. The trust-level is computed by combining trust values from different servers (community-based servers, domain-based servers, and trust-based servers). At the same time, it is not allowed to communicate the community-based servers across domains. The community-based server helps the computation of trust by considering the information of a particular domain while an interaction is taking place among a node and its domain. If any of the nodes are compromised by malicious activity, others will not be affected. However, the HoliTrust Model is theoretical, making it challenging to understand the performance of the model and its effectiveness.

#### 3.5.2. Distributed AA

Distributed AA schemes can solve the centralised AA schemes shortcomings. There is no central entity in distributed environments and the participants work together to develop relation based on mutual interactions. Each entity evaluates and distributes trust expertise to particular nodes autonomously.

The Localized Encryption and Authentication Protocol (LEAP+) employs GWN as a security layer for WSNs focusing on distributed trust systems. Its existence, however, is restricted to particular tasks such as [109] node activation. The sensor nodes work with neighbouring nodes to build and maintain core similarities. The resemblances allow LEAP+ to minimize the overall control of a given point and the overhead connectivity of direct communication between a sensor node and a neighbouring node.

In [110], Ruidong et al. proposed a distributed AA Scheme to allow distributed authorization by using Identity-based Signature for distributed identities verification of the publishing nodes and by using a Ciphertext Policy in the ABAC model. The framework proposed by the authors has three main stages, initializing, publishing date securely, and retrieval of data securely, which seamlessly integrates authentication and authorization into the cloud. As per the experimental findings, the proposed scheme achieved lower bandwidth costs than the existing systems. Likewise, in [111], the authors illustrate in EDTM (Efficient Distributed Trust Model) for authentication to investigate trust and relationship between nodes or systems by considering only the behaviour for AA was not sufficient. Correspondingly, the authors of [112] have developed a preventive maintenance approach for IoT devices, which was presented as a self-directed learning data acquisition unit during the ML algorithm execution. The systematic approach focuses on risks that enable the tracking device to lose data or knowledge. These monitoring methods are also used for various actuators as a virtual system. However, the Bubbles of Trust in [69] also convey the distributed characteristics of IoT authentication.

Generally, distributed AA schemes are more reliable than centralized AA schemes. The authentication and authorization costs may be allocated to participants to increase scalability. Yet, distributed systems are more prone to collusion. The control and analysis of the network become more complicated, and overhead for specific nodes appears to be greater than that in centralized schemes.

From the AA schemes literature review, it has been seen that there is a considerable lack of research for IoT authentication and authorization. However, there is very little AA research conducted based on ML. As the number of IoT nodes increases rapidly, and new unknown threats and attacks will also increase. Therefore, the various security issues need to be resolved as quickly as possible before they can cause any significant damage. ML is promising to solve already known and unknown threats by a learning process. ML-driven schemes can solve several issues of AA to enhance IoT security.

## 4. Open Issues, Challenges, and Future Directions

IoT is considered (a) very complex, with rapid and repetitive shifts in the security specifications of network entities; (b) extremely heterogeneous, with various forms and attributes of network entities. Therefore, various issues need to be addressed before ML-based AA can incorporate into IoT to improve security. A wide variety of encryption methods such as hash key, XOR-based encryption, Elliptic-curve cryptography (ECC) use of a smart card, and biometric technologies are used for a AA process. The purpose of any newly developed AA scheme is to be lightweight and defend IoT nodes from attacks by considering the low processing power factor and the limited space in IoT devices. Since the utilization of IoT devices is rapidly increasing, robust authentication and authorization schemes are needed for individual devices and their services. However, most ML-based Authentication and Authorization schemes have not considered essential requirements for authenticating and authorizing IoT nodes. Hence, the following sections address various concerns and challenges with future research directions for AA related to IoT for the future.

- **Resource Constraint and Robustness of Authentication Protocols:** Sensors are the end nodes that are limited in resources (limited battery capacity, processing resources). The protocols need to be lightweight and move between resource usage and safety. Moreover, the structure for IoT authentication mechanisms, especially for resource-restricted and the environment of IoT framework, should take into account low computation costs. This reinforces the need for the creation of authentication systems to implement lightweight encryption algorithms and conventions. The authentication protocols must have robustness against potential attacks, including Sybil, node capture, interpret, identification of passwords, message breaches, brute forces, broker, protection, collision, and text selection.
- **Authorization for Each Service:** User identities are used to access one or more services, but some of these identities can only be used by a specific service. Therefore, a scheme must provide a mechanism to access the device, depending on the different services that claim to access and use identity data. Thus, user information exchange between different system services is not allowed or should follow the authorization policy of individual services.
- **Datasets Unavailability:** Powerful ML-driven schemes required massive datasets. The performance of different ML algorithms must be evaluated and compared by credible datasets from actual physical environments. The data include personal and vital details that distinguish the individuals and their actions and personality. For example, body area networks (BAN) and other IoT technologies can violate consumer privacy, and smart home data can reveal personal actions and behaviours. It is therefore vital for developers not to jeopardize consumer safety by the data used by ML-driven techniques. To date, the anonymity methods must need to be considered before it is used for analytics as the demonstration showed that the techniques for anonymity are hacked. Training models may be jeopardized by inserting fake data. It may be difficult to gather data while protecting privacy and confidentiality. Questions like how ML algorithms should be implemented, and which degree of privacy ML algorithms can uphold need to be answered. Therefore, it is important to examine data security and user privacy conservation techniques in ML-driven analytics for IoT networks. Actual IoT scenarios cannot completely be encompassed in the data produced by simulations. Production of synthetic data can become quite costly for computing and training.
- **Sharing Information to Reduce Overhead:** The overhead of authentication protocols is a key factor in communication, particularly in resource-restricted devices. Fewer messages should be shared between communication partners. Owing to the limited capacity of IoT devices, the volume of the message should be as low as possible.
- **Trust Relationship among Services:** IoT security system needs to be flexible as it has to support multiple nodes and attach additional nodes. All three layers of the IoT system (application, communication, and perception layer) will provide authentication service. The user can have and use different identities to access more IoT services. Therefore, the AA must introduce devices in the authentication phase using a precise selection of identities automatically or semi-automatically. The trust management scheme must manage multiple relationships to select the identities required in the context of access to the service. Therefore, the challenge of authenticating multiple shared devices is expressed by a single user who uses more than one service at a time. The problem can be solved by SSO in SAML, OAuth, etc. The use of devices shared by different users is another important aspect. It is currently addressed through the so-called “sandbox” techniques to differentiate users in the IoT environment. A D2D authentication function is represented as the authentication of multiple devices in an IoT network. An application user device can access all its devices and individual services after performing initial authentication on a single device and authorized access services through trust relationships among related devices. Access to a connected device will allow the collection of contextual metadata and sensor node data in centralized services management.
- **Easy, Time & Location Driven Authentication:** Throughout the design of IoT authentication frameworks that enable several applications to be accessed from one single sign-on mechanism and thus minimize user interface repetition, the complexity of the application throughout IoT networks must be addressed. Nevertheless, the machine must learn about the ownership of the device and the operator to whom it is linked to enabling efficient contact with this program. Alternatively, it is necessary to reply promptly to the sender, as authentication needs to emerge on time. Protocols often use timestamps and session keys to secure them from attacks. However, the physical context, such as the device’s physical information, temperature, location, behavioural analysis of IoT node, etc., should also need to be considered in developing Context-Aware ML-based AA schemes. Context-awareness in ML-based AA can provide a more robust authentication and authorization and defend against attacks by using the context as AA factor have been discussed in [113]. Moreover, IoT security should rely on centralized and distributed characteristics that consider timing.
- **New & Strong Authentication Schemes:** Researchers use XOR-ing and hashing algorithms to render the authentication protocol lightweight. Additionally, protocols require numerous guarantees to maintain authenticated data retention. There are also other opportunities to implement modern authentication methods based on ML. The open issues in IoT security are quantum computation, quantum bit interaction, and quantum cryptography. Considering the attacks in IoT networks are massive, especially at the first step in network access, specific network packets are critical for that device efficiency through their behaviour. Therefore, it is necessary to establish trust in each node to protect the IoT network from all possible attacks using a robust ML-based authentication protocol.
- **Anonymity:** Anonymity is a significant issue in IoT due to extensive data sharing. An attacker may target the IoT network to collect details regarding IoT nodes, which would reveal critical information, e.g., medical records. Alternatively, an attacker can track down a user or object’s location and harm the devices or device properties, especially in a mobile network. Future work may focus on data anonymization and develop an IoT security process, which illustrates transparency.

As from the literature, none of the reviewed schemes is cross-layered to fit under the scope of the Cyber-Physical domain for IoT. There is an urgent need for such authentication schemes to mitigate attacks across different IoT architecture layers where IT and OT overlap, blurring the line for defining AA schemes. ML algorithms such as RL and adversarial ML are the most promising algorithms that can defend against dynamic threats and attacks without changing or customizing IoT AA functions or human intervention.

- **RL for AA**: The AA frameworks incorporate artificial learning techniques, such as enhancement of learning and game theory, for understanding and identifying hostile actors through initialization and connectivity, and taking choices regarding protection with greater consistency in a complex operational environment. The operating environment dynamics guarantee different policies (or collections of activities) to optimize node access in every IoT setting. The Markov decision-making processes (MDPs) initially established RL. An agent in RL explores how their behaviors impact the world through trial and error. RL aims to produce long-term outcomes that can be used to answer increasingly technical problems that traditional methods do not address. It is the best option where no training databases are accessible, and learning is done by practice and external contact. In a static stochastic framework, the RL enables a single entity to learn a strategy that maximizes possible late rewards. However, as many agents implement the information sharing process in a collaborative context, it may go beyond the MDP pattern. The optimal strategy in these structures is based not just on the context but also on the other actor’s policies. In this particular instance, game theory is widely used.
  Nevertheless, RL is afflicted by a dimensionality constraint, and its use in the actual physical environment is constrained. RL also needs data to learn from experience and requires significant computing resources. It is combined with other ML strategies such as Deep Reinforcement Learning (DRL) to address RL shortcomings. In comparison, given the limited amount of data accessible, both RL and DRL algorithms can do better in AA than most ML algorithms. Moreover, the RL can learn from the environment, which will forward the RL-based AA schemes to fight against current threats and upcoming threats.
  Similarly, malicious nodes may also implement ML methods and initiate attacks during the initialization of the AA process. Thus, in the IoT environment, further studies are needed to be anticipated into the usage of ML in the AA framework and in resolving ML-based attacks.
- **Adversarial ML**: Most of the ML algorithms are subject to adverse attacks irrespective of the category of the algorithm utilized. The more general inconsistency between an intruder and a protector, an ML-led scheme is designed to protect from attacks [114]. Nonetheless, all schemes are susceptible to both observational and cause-effective attacks.
  The intruder can affect the ML algorithms training or testing by altering data or a pattern. An intruder applies reverse engineering primarily based on a scheme identification and then exploiting it to avoid detection. Both of these cases can also be launched for IoT protection at the same time. In general, ML-driven schemes involving retraining the scheme very frequently or require the latest data are often more likely to be targeted in these categories. For example, the ML framework dataset can be rendered to various outcomes by slight modifications (e.g., adding a minimal quantity of noise while maintaining the data identified with the actual PHY-Layer signal).
  Furthermore, it remains vital to build resilient, robust, and reliable ML-driven schemes to cope with complex attacks by intelligent opponents. Adversarial ML analysis requires further attempts to track by adversaries and malware skipping attacks. ML-driven AA systems are prone to attacks of poisoning. An attacker could inject malicious information into an ML training dataset. The learning process is badly impaired in this situation. Therefore, a weak paradigm is enlightened with the learning process. It should be remembered that collusion attacks can be used as an initial step before malware skipping attacks are later initiated. There is also a gap that an intruder might manipulate with the training. An intruder can particularly abuse the vulnerability of different underlying ML algorithms. To illustrate, an Intruder might generate malicious information throughout a malware skipping attack, which purposely causes system errors in the ML algorithm.
  As a result, these attacks make the system vulnerable in scenarios such as (a) when initial data filtration process is unsuccessful, the intruder may utilize the data which vary considerably from the actual data which will generate the unpredictable actions by ML algorithms; and (b) where the data needed to train the scheme are not always adequate for the function underlying an ML-driven scheme to achieve the ideal learning process [115].

## 5. Conclusions

IoT is proliferating rapidly in different areas. This raises serious concerns on the security of the IoT devices due to recent attacks on the IoT network, showing how vulnerable are IoT networks. If not secured appropriately, IoT networks can increase the vulnerabilities in the existing IoT network and seriously threaten end-users. The shortcomings can be minimized based on a comprehensive analysis of IoT networks in terms of authentication and authorization and their threats as traditional and new methods change and improve.

This review provided a comprehensive review and proposed a detailed taxonomy of AA in IoT networks. Based on the taxonomy, we discussed various aspects of AA with general and ML-driven techniques to analyze how AA can improve IoT ecosystem security and identify potential research opportunities. IoT architecture with respect to AA schemes is also discussed, focusing on various attacks and threats in each IoT layer. The requirements and current issues have also been discussed for IoT applications using ML algorithms for AA. However, in most ML-based IoT AA schemes, the researchers have not considered common performance metrics such as the delay and the location for the authentication of IoT nodes. Furthermore, there are very few ML-driven authorization mechanisms available in the literature. Hence, it is crucial to investigate ML-driven AA schemes by considering standard and ML performance metrics to improve IoT environment security, including the perception layer, communication layer, data processing, analysis layer, and application layer. Besides, architectural approaches such as using a hybrid approach instead of either centralized or only distributed approach can be used along with ML for IoT AA.

Finally, the paper introduces future research direction by utilizing ML-based AA to tackle IoT security problems in a collaborative and unified way. For example, the wide variety of encryption methods used for an AA process can be re-designed to be lightweight, collaborative, and flexible using ML-based approaches to fulfill the critical requirements for IoT security. Several issues like the robustness of authentication protocols, per service authorization, datasets unavailability, overhead reduction using information sharing, services-trust relationship, easy, time, and location-driven authentication schemes, and Reinforcement Learning for AA and Adversarial ML were discussed in this paper.

## Figures and Tables

**Table 1:** Existing Review on AA Schemes.

References	Authors Contribution(s)	Limitation(s)
Yang et al. [16]	Discussing IoT attacks, surveying the most critical limitations of IoT devices and their solutions, analyzing the security issues in different IoT layers, and exploring IoT access control schemes and architectures.	The survey mainly focuses on battery life and computational lightweightness without any taxonomy, while other essential IoT security requirements are neglected.
Lin et al. [17]	Overview of the safety and privacy problems, and challenges of fog/edge computing and IoT applications.	Available security and privacy control schemes are neglected in this survey. Moreover, the requirements are not specified appropriately.
Xiao et al. [18]	Addressing the importance of the ML-driven methods for IoT security and privacy by showing the implementation of ML-driven security solutions for IoT networks.	Lacks taxonomy and little discussion on the attack detection schemes.
Hajiheidari et al. [7]	Discussing the security issues in the IoT environment and categorize the types, characteristics, attacks and respective simulation, and the theoretical solution by illustrating the advantages and disadvantages of the selected mechanisms.	Focuses on IDS in IoT without emphasis on vital IoT security requirements and a minimal discussion on AA.
El-hajj et al. [15]	This paper gives a near-complete and up-to-date view of the IoT authentication field. It provides a summary of a large range of authentication protocols proposed in the literature.	A limited number of related works considered. Moreover, the author covered generic authentication schemes for the survey.
Preeti et al. [19]	This paper explored the viability of ML-driven schemes to identify intruders in IoT networks by applying these methods in intrusion detection systems, either by irregularities or traffic classification	The IoT security requirements are not appropriately considered. The metrics required to measure ML-based IDS performance are not presented.
Hussain et al. [20]	Discussing in-depth protection and privacy analysis of the layers (physical, network, and application). Extensively describe shortcomings of the current ML-driven approaches.	IoT attacks are not specified systematically and metrics needed to measure ML-based security schemes are not discussed.

**Table 2:** Benefit(s) and Limitations of General Authentication schemes.

References	Benefit(s)	Limitation(s)
Jan et al. [66]	A centralized lightweight key-based Authentication scheme over CoAP. Able to detect DoS and eavesdropping attack.	Require more than four message exchange before establishing communication. Moreover, the end-to-end delay is not considered for benchmarking the performance, and it is not sufficient to tackle Sybil, MiTM, node capturing, and flooding attack.
Hamidi et al. [68]	A heterogeneous biometric authentication system that considered privacy profiling and tracking over the application layer.	Attacks are not considered, and performance metrics have not been properly considered for benchmarking. Moreover, the analysis of results is not adequately shown.
Zhou et al. [70]	A lightweight two-factor authentication scheme using RFID tag with the one-way hashing and XOR-ing in the cloud computing environment. Moreover, costs and computational efficiency are also considered and demonstrated for the low-resource environment.	The cost of communication and the computation cost at the cloud are higher. Moreover, the attacks are not considered during authentication.
Karthikeyan et al. [79]	A lightweight, energy-efficient authentication scheme for a centralized IoT environment using X.509 PKI certificates is presented.	The practical analysis of security and scalability requirements for PKI-based IoT authentication systems are not considered. Moreover, attacks are not considered, and performance metrics have not considered properly for benchmarking.
Gope et al. [83]	A scalable, lightweight Authentication scheme with privacy profiling and tracking for IoT application layer is presented. A PUF key is shared as security credentials and provide tolerance against DoS and eavesdropping attacks. Moreover, the cost of communication and storage is minimal.	The end-to-end delay is not considered for benchmarking the performance, and it is not sufficient to tackle Sybil, MiTM, node capturing, and flooding attack.
Alizai et al. [67]	A lightweight multi-factor authentication using digital signature and device capacity is presented. It can be utilized to authenticate both the end node and the server node from the application layer and physical layer perspective. Able to defence against MITM attacks and replay attacks.	The server node validates the signatures and the outcomes from the function during device authentication. At the same time, DoS/DDoS, Sybil attacks can take advantage, and considerable performance metrics are not discussed.
Hammi et al. [69]	A blockchain-driven new authentication scheme for IoT systems aims to create bubbles by dividing devices into virtual zones to identify and trust each other through grouping.	To validate a transaction, this consensus protocol takes a considerable amount of time which is not feasible for real-time applications. Moreover, the transaction fee in the public blockchain is considered to be inefficient.
Haroon et al. [50]	An enhanced lightweight authentication scheme over DTLS by sharing a pre-shared key between the server and the trusted third party (TTP) is discussed. It uses NHC and IPHC as compression schemes. As a result, it decreases DoS attack and computational overhead.	Despite the benefits of reducing DoS attacks and computational overhead compared to other Lithe and DTLS methods, the multiple create requests and multiple handshake requests from various requesters can cause drainage problems, as it needs to control numerous computations.

**Table 3:** Summary of Recent Studies on General IoT Authentication Schemes.

Reference		[66]	[68]	[70]	[79]	[83]	[67]	[69]	[50]
**Requirement**	Lightweightness	✓		✓	✓	✓	✓		✓
	Privacy Profiling and Tracking		✓			✓		✓	
	Heterogeneity		✓					✓	
	High Availability							✓	
	Dynamic Registration	✓						✓	
	Scalability					✓		✓	
	Fault Tolerance		✓			✓	✓	✓	✓
	Early Detection of Attacks							✓	
	Resilience and robustness	✓		✓		✓		✓	
	Energy-Efficiency			✓				✓	✓
	Deployment Flexibility								
**Layer**	Application	✓	✓		✓	✓	✓	✓	
	Network								✓
	Physical			✓			✓		
	Perception								
**Token**	User Credentials				✓		✓		
	Key	✓	✓	✓		✓			✓
**Procedural**	Single					✓			
	Dual	✓		✓	✓			✓	
	Triple								
**Characteristics**	Centralized	✓		✓	✓	✓	✓		✓
	Distributed		✓					✓	
	HW-driven			✓		✓			
	Certificate-driven				✓				
	Blockchain-driven							✓	

**Table 4:** Summary of Recent Studies on General IoT Authentication Schemes Performance metrics and Attacks.

Reference		[66]	[68]	[70]	[79]	[83]	[67]	[69]	[50]
**Evaluation metrics**	Average Response Time	✓						✓	✓
	Handshake duration	✓						✓	
	Average memory consumption	✓							
	End-to-End delay								
	Impact on Throughput								
	Ratio of Packet delivery								✓
	Cost of communication			✓		✓			
	Cost of computation			✓					
	Cost of storage/memory		✓			✓			
	Energy-Consumption			✓				✓	✓
**Attacks**	DoS/DDoS	✓				✓		✓	✓
	MiTM						✓		
	Spoofing							✓	
	Sybil							✓	
	Off-Line Guessing			✓				✓	
	Forgery			✓				✓	
	On-Off							✓	
	Eavesdropping	✓				✓			

**Table 5:** Benefits with Performance Accuracy and Limitations of different ML algorithms.

Techniques	Benefit(s) with Performance Accuracy	Limitations(s)
Q-Learning [24,25,62,90,91,94]	Able to detect Jamming, Spoofing, Eavesdropping, DoS/DDoS, Malware attacks using optimal authentication threshold while the mis-detection of spoofing minimized by 61.72% and false positive by 93.33%.	Classfication Accuracy, Miss-detection, False/True Positive, False/True Negative has not analyzed. Morever DoS/DDoS, Jamming and Malware attacks have not been formulated.
KNN [88]	Detects Intrusion attacks. Accuracy has not been specified.	FAR, FRR, EER, False/True Positive, False/True Negative have not analyzed. Moreover, only designed to detect intrusion from sequential request.
SVM [63,95]	Detects Intrusion attack and also able to classify Spoofing attack. Accuracy has not been specified.	Only algorithms, strategies, and application discussed with limited analysis on spoofing.
Naïve Bayes [95]	Detects Intrusion attack. Accuracy has not been specified.	
Dyna-Q [24,62]	Detects Intrusion attack. Moreover, the mis-detection rate of Dyna-Q is 6.9% lower and false positive rate is 5% lower than Q-Learning.	The applications are Limited to Malware Attack only.
DQN [93]	Detects Jamming attack. Accuracy has not been specified.	The attack design Limited to Jamming without analyzing FAR, FRR, EER, False/True Positive, False/True Negative metrics.
DNN [92]	Detects Spoofing attack with accuracy more than 94.5% and user identification accuracy is 93%.	No considerations on low-resource devices and limited to WiFi enabled devices.
Distributed Frank Wolfe [61]	Detects Spoofing attack and saves communication overhead by 37.4%.	DoS/DDoS, Eavesdropping attacks have not been considered to be formulated.
Incremental Aggregated function [61]	Detects Spoofing attack and saves the computational overhead by 71.3%.	DoS/DDoS, Eavesdropping attacks have not been considered to be formulated.

**Table 6:** Summary of Recent Studies on Machine learning approaches for Authentication with Performance.

		Techniques																
		Q-Learning [90]	KNN [88]	Nonparametric Bayesian [89]	Multivariate correlation analysis [86]	SVM [95]	Naïve Bayes [95]	Q-Learning [62]	Dyna-Q [62]	SVM [63]	Q-Learning [25]	Q-Learning [94]	Q-Learning [91]	DQN [93]	DNN [92]	Q/Dyna-Q/PDS [24]	Distributed Frank-Wolfe [61]	Incremental aggregated gradient [61]
**Performance** **Metric**	Detection Accuracy		✓		✓	✓	✓	✓	✓	✓		✓			✓	✓	✓	✓
	Root Mean Error		✓		✓	✓	✓					✓						
	Energy Consumption	✓											✓	✓				
	SINR	✓											✓	✓				
	Average Error							✓	✓	✓					✓		✓	✓
	Classification Accuracy		✓			✓	✓	✓	✓	✓					✓	✓	✓	✓
	False Alarm		✓					✓	✓	✓					✓		✓	✓
	Miss-Detection							✓	✓	✓					✓		✓	✓
	False/True Positive															✓		
	False/True Negative															✓		
	Detection Latency															✓		
	Proximity latency			✓							✓							
	Data Secrecy			✓							✓							
**Attacks**	DoS				✓							✓						
	Jamming	✓											✓	✓				
	Spoofing							✓	✓	✓					✓		✓	✓
	Intrusion		✓			✓	✓											
	Malware															✓		
	Eavesdropping			✓							✓							

## List of Acronyms and Abbreviations

**Acronyms**	**Abbreviations**
AA	Authentication & Authorization
ACL	Access Control List
AES	Advance Encryption Standard
AI	Artificial Intelligence
ABAC	Attribute Based Access Control
BAN	Body Area Network
BP	Back-Propagation
CapBAC	Capability-Based Access Control
CA	Certificate Authorities
CRP	Challenge Response Pair
CIR	Channel Impulsion Responses
CSI	Channel State Information
CoAP	Constrained Application Protocol
XSS	Cross-Site Scripting
DTLS	Datagram Transport Layer Security
DNN	Deep Neural Network
DoS	Denial of Services
DDoS	Distributed Denial of Services
ERR	Equal Error Rate
XMPP	Extensible Messaging and Presence Protocol
FAR	False Acceptance Rate
FRR	False Rejection Rate
DFW	Frank-Wolfe
GWN	Gateway Node
IAG	Incremental Aggregated Gradient
IGMM	Infinite Gaussian Mixture Model
I2C	Inter-integrated Circuit
IMT	Internet of Medical Things
IoT	Internet of Things
ID	Intrusion Detection
IPHC	IP Header Compression
LEAP+	Localized Encryption and Authentication Protocol
MIN	Machine Identification Number
ML	Machine Learning
MITM	Man in the Middle
MDP	Markov Decision-Making Processes
MAC	Media Access Control
MQTT	Message Queuing Telemetry Transport
MLP	Multi-Layer Perceptron
NB	Naive Bayes
NHC	Next Header Compression
PSO	Particle Swarm Optimization
PHY-Layer	Phyisical Layer
PKI	Public Key Infrastructure
QoS	Quality of Services
RSS	Received Signal Strength
RSSI	Received Signal Strength Indicators
RL	Reinforcement Learning
RBAC	Roles Based Access Control
SMQTT	Secure Message Queue Telemetry Transport
SAML	Security Assertion Markup Language
SNEP	Sensor Network Encryption Protocol
SNSP	Sensor Network Security Protocols
SPI	Serial Peripheral Interface
SSO	Single Sign-On
SVM	Support-Vector Machines
TLS	Transport Layer Security
TTP	Trusted Third Party
WoTs	Web of Things
WSN	Wireless Sensor Network
